# Direct and social genetic parameters for growth and fin damage traits in Atlantic cod (*Gadus morhua*)

**DOI:** 10.1186/1297-9686-46-5

**Published:** 2014-01-24

**Authors:** Hanne M Nielsen, Brage B Monsen, Jørgen Ødegård, Piter Bijma, Børge Damsgård, Hilde Toften, Ingrid Olesen

**Affiliations:** 1 Nofima - Norwegian Institute of Food, Fisheries and Aquaculture Research, P.O. Box 210, N-1431 Ås, Norway; 2 Department of Animal and Aquacultural Sciences, Norwegian University of Life Sciences, P.O. Box 5003, N-1432 Ås, Norway; 3 Present address: AquaGen, Department of Animal and Aquacultural Sciences, Norwegian University of Life Sciences, P.O. Box 5003, N-1432 Ås, Norway; 4 Animal Breeding and Genetics Group, Wageningen University, 6709PG Wageningen, The Netherlands; 5 Nofima - Norwegian Institute of Food, Fisheries and Aquaculture Research, P.O. Box 6122, N-9291 Tromsø, Norway; 6 Faculty of Biosciences, Fisheries and Economy, University of Tromsø, N-9037 Tromsø, Norway

## Abstract

**Background:**

The aim of the study was to estimate genetic parameters for direct and social genetic effects (SGE) for growth and welfare traits in farmed Atlantic cod (*Gadus morhua*). A SGE refers to the effect of an individual’s genes on trait performance of its social partners. In total, 2100 individually tagged juveniles from 100 families at an average age of 222 days post-hatching were used. Each family was separated into three groups of seven fish, and were randomly assigned to 100 experimental tanks, together with fish from two other families. Body weight and length of the first, second and third dorsal fin and the caudal fin measured by digital image analysis were measured at the start of the experiment, after two weeks, and after six weeks. Fin erosion was scored subjectively after six weeks. Variance components estimated using a conventional animal model were compared to those of an animal model including a SGE.

**Results:**

Heritabilities from the conventional animal model ranged from 0.24 to 0.34 for body weight and 0.05 to 0.80 for fin length. Heritabilities for fin erosion were highest for the first dorsal fin (0.83 ± 0.08, mean ± standard error) and lowest for the third dorsal fin (0.01 ± 0.04). No significant SGE were found for body weight, whereas SGE for fin lengths were significant after two and six weeks. Contributions to the total heritable variance were equal to 21.5% (6.1 ± 2.1) for the direct effect, 33.1% (9.4 ± 3.2) for the direct-social covariance, and 45.4% (12.9 ± 4.1) for the social variance for length of the first dorsal fin. For fin erosion, SGE were only significant for the second and third dorsal fin.

**Conclusions:**

Including SGE for fin length and fin erosion in the animal model increased the estimated heritable variation. However, estimates of total heritable variances were inaccurate and a larger experiment is needed to accurately quantify total heritable variance. Despite this, our results demonstrate that considering social breeding values for fin length or fin erosion when selecting fish will enable us to improve response to selection for welfare traits in Atlantic cod juveniles.

## Background

Atlantic cod (*Gadus morhua*) is an omnivorous fish species with cannibalistic and aggressive behaviour [1,2]. Aggressive interactions between fish may in general cause fin damage, including splitting, erosions, and thickening of the fins [3]. Fin damage may also affect growth, survival and the general welfare of the fish [3,4], and fin damage assessments may be used as an indicator of the strength of social hierachies [5]. It is however largely unknown to which extent the social interactions between fish have a genetic component. Atlantic cod is a relatively new farmed species in Norway. Selective breeding programs for cod have until now mainly focused on improving growth rate, and there is currently little knowledge about the size of the heritable genetic components of welfare-related traits such as aggression.

The general idea behind socially affected traits is that the observed phenotype of an individual does not only depend on the genes of the animal but also on the genes of the other animals in the group [6-9]. Thus, each animal may have a direct genetic effect on its own phenotype but also a social genetic effect (SGE, also known as associative effect or indirect genetic effect) on the other animals in its environment. In classical breeding, the models for breeding value estimation only account for the direct effect of the animal itself, ignoring social effects. Studies in other species such as quail [10], pigs e.g. [11] and poultry, e.g. [12,13], have shown that social interactions between animals may be responsible for a substantial part of the heritable variance for traits such as growth and survival. For example, Ellen et al. [12] found heritabilities of 2 to 10% for survival in three layer lines when using a conventional animal model, whereas the total heritable variation ranged from 6 to 19% when both direct and SGE were considered. Thus, taking into account SGE can increase response to selection for traits affected by social effects. Genetic correlations between direct and social effects are also important, since the presence of SGE may reverse response to selection if SGE are ignored when selecting breeders to produce the next generation [6,10,14].

Little is known about SGE in farmed aquaculture species. In mussel (*Mytilus galloprovincialis*), Brichette et al. [15] studied growth cultures and estimated the genetic correlation between direct and social effects of −0.2. Heritabilities including direct effects only for shell length at an age of around eight and eleven months were 0.104 and 0.232, whereas the proportions of phenotypic variance described by social effects were only 0.010 and 0.087. In Nile tilapia (*Oreochromis niloticus*), there is one study on SGE of body weight [16]. However, these authors did not find any indications of SGE of body weight in a six-week experiment.

The aim of this study was to estimate direct and social genetic parameters for growth traits and traits related to fish welfare (fin damage) in Atlantic cod juveniles. Data were analyzed using both a classical animal model and an animal model with direct and social genetic effects in order to compare the estimated total heritable variance of the two models.

## Methods

### Fish material and experimental design

In total, 2100 cod juveniles from 100 half- and full-sib families (73 sires and 100 dams) that originated from the third generation of the Norwegian Cod Breeding Program in Tromsø were used. These fish have mainly been selected for growth and to some extent for resistance to vibriosis (see Bangera et al. [17] for more details about the breeding program). The complete pedigree contained 160 sires and 197 dams. The fish were hatched in March 2009 and each family was kept in separate tanks until tagging. In September, the fish (average weight approximately 24 g) were anaesthetized (metacaine, 0.08 g/L) and tagged with passive integrated transponders (PIT-tag ID100A, Trovan Ltd, Hessle, UK) injected into the abdominal cavity. From tagging until the start of the experiment in November, the fish from each family were held in one common 500 L tank and under similar conditions. The experimental design, including the number of fish and families per tank needed to obtain reliable estimates of genetic effects, was determined by simulations and power calculations (see [18]). In this study, the fish were not subjected to any potentially harmful treatment, and we did not sample biological material such as blood samples. Consequently, the local representative approved the experiment without any application according to the rules by The Norwegian Animal Research Authority at the time the experiment was conducted.

At the start of the experiment (2 November 2009), each of the 100 families with 21 fish was divided into three groups of seven fish. The three groups from each family were then randomly assigned to one of 100 tanks of 130 L, so each tank contained 21 fish from three families, giving an average density of fish of 5.6 kg/m^3^ at the start of the experiment. At the end of the experiment, the average density was about 10.3 kg/m^3^. During the experimental period, the fish were fed a dry feed (Classic Marine Biomar) on a restricted basis (60% of normal feeding level using a feed conversion ratio of 1.5) 12 times per day, in order to promote social interactions among the fish. The feed was provided to each tank using an automatic feeder and the amount of feed for each tank was calculated based on feeding rate and biomass in each tank after two weeks. Each tank was supplied with unfiltered sea water (30–34% salinity). Water temperature was recorded daily and oxygen content was recorded twice a week in the tanks with the highest biomass and once a week in the other tanks. The mean water temperature was 8.7°C (range 8.2-9.8°C) and the light regime in the housing facilities was continous. Water flow was adjusted to 5 L/min during the whole period, securing levels of oxygen saturation of between 87 and 97% throughout the experiment. A circumferential water current of about 5.6 cm/s was created by directing the water inflow through vertical, perforated inlet pipes, as described in [19]. The average length of the fish increased from 15.4 cm at the start of the experiment to 18.4 cm at the end of the experiment, and consequently, the water velocities corresponded to relative speeds that decreased from approximately 0.36 body lengths per second (BL/s) at the start to 0.33 BL/s at the end of the experiment.

### Recordings

The experiment lasted for six weeks and recordings of the fish were performed three times. Recording 1 was conducted on 2–3 November 2009, when the fish were stocked into the experimental tanks. Recording 2 was performed two weeks after the start of the experiment and recording 3 at the end of the experiment. Before each recording, the fish were anaesthetized with metacain (MS-222, 0.08 g/L), after which body weight (0.1 g) and length (0.1 cm) of the fish were measured. In addition, erosions of the first, second and third dorsal fins and of the caudal fin were scored subjectively at the end of the experiment. Fin erosion is damage to the fin that results in loss of epithelial fin tissue and all or part of the fin ray [3]. The degree of fin erosion was scored by a single person on a scale from 0 to 100%, in 5% intervals. At recording 1, 20 fish died due to an accident during sedation. These 20 fish were replaced with fish from the same families as those of the fish that died. The number of fish that died during the whole experiment was also recorded.

### Measurements of fin length using digital image analysis

**Figure 1:**
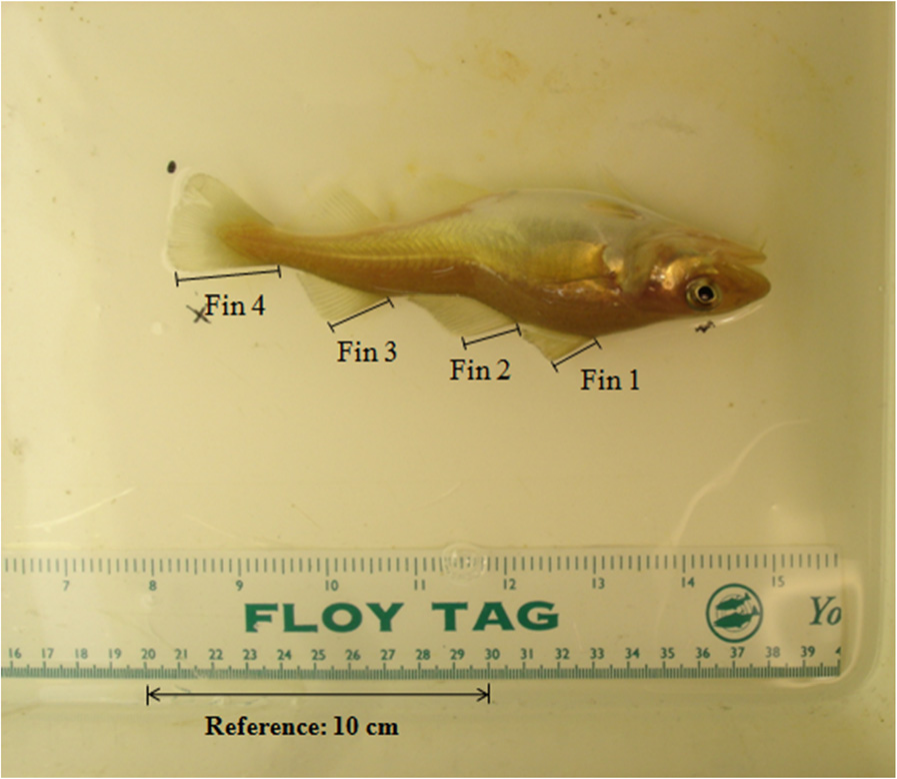
Location of the measurements taken for maximum fin length of the three dorsal fins and the caudal fin.

In order to measure the length of the fins, a digital photo was taken of each fish at each of the three recordings. Before the photo was taken, the fish was placed on a uniform white background with the left side of its body up. A calibration ruler and two pieces of paper with the tank number and the number of the fish were placed above and beside the fish. Using digital image analysis (MATLAB software version 7.12, r2011a), lengths of the three dorsal fins and of the caudal fins were measured by estimating the maximum length of the fin (i.e. parallel to the fin rays). A ten cm scale was used as a calibration vector (see Figure 1). The position of the cursor and mouse clicks were used to measure the fin lengths by locating the starting points of fins on the base side and the end points of the fins on the outer side, along with the fin ray. The measurements of the length of the four fins were done by three persons, each scoring 40, 49 and 11% of the fins.

### Studied traits

Body weights at the three recordings, specific growth rate (SGR), change in condition factor (CCF, between recordings 1 and 3), fin length, and fin erosion were considered for statistical analysis. The condition factor is an expression of the condition of the fish based on the assumption that weight is proportional to the cube of the length of the fish, such that the condition factor is higher when fish are more spherical. CCF from recording 1 to recording 3 was calculated as:

$$\mathrm{CCF}=CF_{3}-CF_{1},\;\mathrm{where}\;CF_{i}=\frac{\mathrm{weigh}t_{i}}{{\left(\mathrm{lengt}h_{i}\right)}^{3}}$$

SGR, defined as percentage increase in body weight per unit time, was calculated as:

$$\mathrm{SGR}=\frac{\ln\left(\mathrm{weigh}t_{3}\right)-\ln\left(\mathrm{weigh}t_{1}\right)}{\left(t_{3}-t_{1}\right)}\times 100\%,$$

where weight1 and weight3 are the weights at recordings 1 and 3 and t_3_ – t_1_ is the number of days between recordings 1 and 3.

Fin damage was quantified based on lengths of the first, second, third and the dorsal fins at each of the three recordings and based on subjective scores of erosion on the four fins at the end of the experiment.

### Statistical analysis

Genetic analyses of the studied traits were performed using the ASReml software [20]. Data were first analyzed using an animal model without social effects:

$$y=\mathrm{Xb}+Z_{D}a_{D}+\mathrm{Wc}+\mathrm{Wt}+e,$$

where **y** is a vector of phenotypes for the observed trait, **b** is a vector of the age of the fish at each recording and the fixed effect of the person (1, 2, 3) who scored fin length (included only when fin length was analyzed), **a**_**D**_ is a vector of random direct additive genetic effects, **c** is a vector of common environmental tank effects in the rearing period, **t** is a vector of experimental tank effects, **e** is a vector of residuals, and **X**, **Z**_**D**_, and **W** are incidence matrices.

The model with social interactions included both the direct genetic effect of the focal individual and the SGE of each of its group mates, as proposed by Muir [10] (see also [14,21]). The model was:

$$y=\mathrm{Xb}+Z_{D}a_{D}+Z_{S}a_{S}+\mathrm{Wc}+\mathrm{Wt}+e,$$

where **a**_**S**_ is a vector of random social additive genetic effects, **Z**_**S**_ is the associated incidence matrix, and the other parameters are as described above. Note that fitting effects of the experimental tank is equal to fitting a social environmental effect for each animal [18].

Based on the above models, the following variance components and parameters were estimated:

$\sigma_{A_{D}}^{2}$ = direct additive genetic variance;

$\sigma_{A_{S}}^{2}$ = social additive genetic variance;

$\sigma_{A_{\mathrm{DS}}}$ = direct-social additive genetic covariance;

$\sigma_{c}^{2}$ = variance of common environmental tanks effects in the rearing period (1, …, 100);

$\sigma_{t}^{2}$ = variance of experimental tank effects (1, ...., 100).

From these estimated parameters, the following parameters were derived:

$\sigma_{\mathrm{TBV}}^{2}$ = variance of the total breeding values;

$\sigma_{P}^{2}$ = phenotypic variance;

T^2^ = the total heritable variance relative to the phenotypic variance: $T^{2}=\sigma_{\mathrm{TBV}}^{2}/\sigma_{P}^{2}$.

The total heritable variance was calculated as:

$$\sigma_{\mathrm{TBV}}^{2}=\sigma_{A_{D}}^{2}+2\left(n-1\right)\sigma_{A_{\mathrm{DS}}}+{\left(n-1\right)}^{2}\sigma_{A_{S}}^{2},$$

where *n* is the number of fish in the tank. This is the total heritable variance, due to both direct and SGE, that determines the potential of the population to respond to selection [22]. Phenotypic variance was calculated as:

$$\sigma_{P}^{2}=\sigma_{A_{D}}^{2}+\left(n-1\right)\sigma_{A_{S}}^{2}+\sigma_{c}^{2}+\sigma_{t}^{2}+\sigma_{e}^{2}.$$

This expression defines phenotypic variance for groups that consist of unrelated individuals.

With SGE, phenotypic variance depends on relatedness between interacting individuals [11], which may hamper the comparison of genetic parameters, such as heritabilities, between studies. Therefore, phenotypic variance was expressed for the “default” situation, i.e., for a population that consists of unrelated individuals.

Heritabilities were estimated using the univariate linear animal models described above. The importance of including the SGE was tested using log likelihood tests by comparing the differences in the likelihood between the traditional animal model and the model that included both direct effects and SGE. In addition, correlations between direct and social breeding values for weight at recording 3 and for fin damage traits with significant social effects were estimated using bivariate animal models that contained the same fixed and random effects as described above for the univariate models.

## Results

### Descriptive statistics

The average weight of the fish increased from 34.5 to 63.5 g (Table 1) during the experiment, resulting in a SGR of 1.45% per day. The length of all four fins also increased during the experiment. The highest level of fin erosion (23.1%) was measured on the first dorsal fin. In total, 28 fish died during the experiment, of which seven died before the second recording, 13 during the second recording and the remaining in the period between recordings 2 and 3. The number of fish that died during the experiment was randomly distributed over families and tanks.

**Table 1:** Descriptive statistics for weight (g) and length (cm) of the fish and fin length (cm) and fin erosions (%) at three recording times

	**Recording 1**		**Recording 2**		**Recording 3**	
**Trait (units)**	**n**	**Mean (s.d)**	**n**	**Mean (s.d)**	**n**	**Mean (s.d)**
Weight (g)	2091	34.55 (11.7)	2058	42.17 (13.8)	2063	63.55 (20.9)
Length (cm)	2096	15.35 (1.5)	2066	16.22 (1.6)	2018	18.38 (1.7)
Condition factor	1999	0.0092 (0.0009)			2046	0.0099 (0.0012)
Change in condition factor					1999	0.00074 (0.10)
Specific growth rate (%/d)					2046	1.45 (0.51)
*Fin length*						
First dorsal (cm)	1948	1.37 (0.31)	2007	1.53 (0.35)	2030	1.58 (0.41)
Second dorsal (cm)	1948	1.33 (0.20)	2010	1.44 (0.22)	2035	1.55 (0.31)
Third dorsal (cm)	1948	1.49 (0.22)	2010	1.61 (0.23)	2038	1.77 (0.32)
Caudal (cm)	1945	2.34 (0.20)	2010	2.42 (0.29)	2038	2.80 (0.34)
*Fin erosion*						
First dorsal (%)					2062	23.09 (15.20)
Second dorsal (%)					2061	14.32 (9.95)
Third dorsal (%)					2060	10.39 (5.49)
Caudal (%)					2060	13.34 (8.18)

Fin erosions were only scored at the end of the experiment (recording 3); growth rate and change in condition factor were calculated from weight and length recordings.

### Conventional animal model

Using a classical animal model, estimates of heritability for weight were 0.34, 0.33, and 0.24 at recordings 1, 2, and 3, respectively (Table 2). The proportion of the phenotypic variance explained by common environment in the rearing period (σ^2^_C_) was relatively constant from recording 1 to recording 3 (7.5 and 7.6%). The effect of the experimental tank was small, increasing from 0 at recording 1 to 2.1% of the phenotypic variation at recording 3. Estimates of heritability for SGR and CCF were equal to 0.16 and 0.13.

**Table 2:** Genetic parameters and standard errors for growth traits at three recording times using a traditional animal model

**Parameter**^**1**^	**Recording 1**	**Recording 2**	**Recording 3**	**Specific growth rate**	**Change in condition factor**
	**Weight**	**Weight**	**Weight**		
σ^2^_AD_	45.5 ± 20.5	59.4 ± 27.5	101.9 ± 54.8	0.04 ± 0.03	0.001 ± 0.0004
σ^2^_C_	10.0 ± 7.4	12.0 ± 9.5	31.8 ± 20.7	0.02 ± 0.01	0^2^
σ^2^_TANK_	0^2^	1.2 ± 1.5	8.9 ± 4.4	0.01 ± 0.004	0.0004 ± 0.0001
σ^2^_E_	76.7 ± 10.7	109.4 ± 14.4	274.8 ± 29.4	0.19 ± 0.01	0.009 ± 0.0004
σ^2^_P_	132.3 ± 6.8	182.1 ± 9.1	417.4 ± 19.1	0.26 ± 0.01	0.01 ± 0.003
h^2^	0.34 ± 0.14	0.33 ± 0.14	0.24 ± 0.13	0.16 ± 0.10	0.13 ± 0.04

^1^σ^2^_AD_ = direct genetic variance; σ^2^_C_ = variance due to common rearing environment; σ^2^_TANK_ = variance of experimental tank; σ^2^_P_ = phenotypic variance; h^2^ = heritability = σ^2^_AD_/σ^2^_P_; ^2^bounded to zero.

Estimates of heritability for fin length (Table 3) at the three recordings were highest for the first dorsal fin (0.60 to 0.80) and lowest for the second dorsal fin (0.05 to 0.33). There were some differences between estimates of heritability for the three recordings for all four fins. The variance due to common environment in the rearing period was small and non-significant for nearly all recordings and fins. The variance of the experimental tank effect increased between recordings 1 to 3 and explained from 6% (caudal fin) to 36% (second dorsal fin) of the total phenotypic variation at recording 3.

**Table 3:** Genetic parameters and standard errors for fin length using a traditional animal model at three recording times

		**Fin**			
	**Parameter**^**1**^	**First dorsal**	**Second dorsal**	**Third dorsal**	**Caudal**
**Recording 1**	σ^2^_AD_	7.15 ± 2.47	0.23 ± 0.38	2.54 ± 0.47	3.30 ± 0.65
	σ^2^_C_	0.47 ± 0.73	0.44 ± 0.19	0^2^	0^2^
	σ^2^_TANK_	0.20 ± 0.09	0.10 ± 0.05	0.010 ± 0.05	0.21 ± 0.10
	σ^2^_E_	2.35 ± 1.24	3.49 ± 0.22	2.20 ± 0.26	5.11 ± 0.41
	σ^2^_P_	10.18 ± 0.77	4.27 ± 0.16	4.83 ± 0.26	8.62 ± 0.39
	h^2^	0.70 ± 0.20	0.05 ± 0.09	0.52 ± 0.07	0.38 ± 0.06
**Recording 2**	σ^2^_AD_	10.43 ± 1.74	1.60 ± 0.33	1.78 ± 0.66	4.65 ± 0.82
	σ^2^_C_	0^2^	0^2^	0.15 ± 0.22	0^2^
	σ^2^_TANK_	0.75 ± 0.19	0.20 ± 0.07	0.30 ± 0.09	0.004 ± 0.06
	σ^2^_E_	1.89 ± 0.91	3.05 ± 0.21	3.08 ± 0.36	3.79 ± 0.46
	σ^2^_P_	13.08 ± 0.90	4.84 ± 0.20	5.32 ± 0.25	8.45 ± 0.45
	h^2^	0.80 ± 0.08	0.33 ± 0.06	0.33 ± 0.12	0.55 ± 0.07
**Recording 3**	σ^2^_AD_	8.50 ± 2.60	1.49 ± 0.67	2.91 ± 1.02	4.79 ± 0.93
	σ^2^_C_	0.59 ± 0.75	0.10 ± 0.22	0.003 ± 0.29	0^2^
	σ^2^_TANK_	2.46 ± 0.49	3.16 ± 0.54	2.54 ± 0.49	0.69 ± 0.19
	σ^2^_E_	2.60 ± 1.31	3.94 ± 0.37	3.77 ± 0.54	6.09 ± 0.55
	σ^2^_P_	14.16 ± 0.98	8.70 ± 0.56	9.25 ± 0.54	11.58 ± 0.54
	h^2^	0.60 ± 0.16	0.17 ± 0.08	0.31 ± 0.11	0.41 ± 0.07

^1^σ^2^_AD_ = direct genetic variance; σ^2^_C_ = variance due to common rearing environment; σ^2^_TANK_ = variance of experimental tank; σ^2^_P_ = phenotypic variance; h^2^ = heritability = σ^2^_AD_/σ^2^_P_; ^2^bounded to zero.

Estimates of heritability for subjectively scored fin erosion (Table 4) were low for the second and the third dorsal fins and the caudal fin but very high for the first dorsal fin (0.83). This pattern was in reasonable agreement that observed for estimates of heritability of fin lengths (Table 3). The experimental tank explained from 13 (first dorsal fin) to 39% (second dorsal fin) of the total phenotypic variation in fin erosion.

**Table 4:** Genetic parameters and standard errors for fin erosion estimated using a traditional animal model

	**Fin**			
**Parameter**^**1**^	**First dorsal**	**Second dorsal**	**Third dorsal**	**Caudal**
σ^2^_AD_	185.0 ± 30.3	16.63 ± 3.87	0.27 ± 1.07	3.74 ± 4.50
σ^2^_C_	0^2^	0^2^	0.66 ± 1.18	4.81 ± 2.19
σ^2^_TANK_	29.03 ± 5.28	37.58 ± 5.87	8.36 ± 1.37	11.78 ± 2.16
σ^2^_E_	7.56 ± 15.75	43.00 ± 2.65	20.78 ± 0.87	46.69 ± 2.75
σ^2^_P_	221.60 ± 16.15	97.2 ± 6.29	30.07 ± 1.52	67.02 ± 2.93
h^2^	0.83 ± 0.08	0.17 ± 0.04	0.01 ± 0.04	0.06 ± 0.07

^1^σ^2^_AD_ = direct genetic variance; σ^2^_C_ = variance due to common rearing environment; σ^2^_TANK_ = variance of experimental tank; σ^2^_P_ = phenotypic variance; h^2^ = heritability = σ^2^_AD_/σ^2^_P_; ^2^bounded to zero.

### Model with social effects

For all growth traits, the log likelihood tests between the model with and without SGE were non-significant (Table 5; the analyses for weight at recording 1 did not converge). This suggests that the conventional animal model is the most relevant model for growth traits and that social effects either do not exist, or their effects are too small to be detected in our experimental design.

**Table 5:** Estimates and standard errors of parameters from a model with social genetic effects for growth traits

**Parameter**^**1**^	**Weight at recording 2**	**Weight at recording 3**	**Specific growth rate**	**Change in condition factor**
LogL	0.97 (0.38)	1.45 (0.23)	0.06 (0.94)	0.91 (0.20)
σ^2^_AD_	54.4 ± 26.7	101.3 ± 53.9	0.03 ± 0.03	0.001 ± 0.0004
σ^2^_AS_	0.02 ± 0.03	0.15 ± 0.12	1.9 × 10^-5^ ± 6.5 × 10^-5^	3.0 × 10^-6^ ± 2.9 × 10^-6^
σ_ADS_	0.34 ± 0.5	0.19 ± 1.29	1.4 × 10^-4^ ± 7.5 × 10^-4^	5.3 × 10^-6^ ± 2.5 × 10^-5^
σ^2^_C_	11.79 ± 9.35	27.33 ± 19.66	0.02 ± 0.01	0^2^
σ^2^_TANK_	0^2^	1.40 ± 6.11	0.01 ± 0.005	0.0003 ± 0.0002
σ^2^_TBV_	76.77 ± 31.53	169.0 ± 77.9	0.04 ± 0.03	0.002 ± 0.001
σ^2^_P_	178.7 ± 8.90	408.0 ± 19.35	0.26 ± 0.01	0.01 ± 0.004
r_AS,AD_	0.31 ± 0.54	0.05 ± 0.33	−0.25 ± 0.58	−0.08 ± 0.38
T^2^	0.43 ± 0.17	0.41 ± 0.19	0.16 ± 0.15	0.22 ± 0.12

^1^LogL = change in Log likelihood relative to the traditional animal model; P-values are given in parentheses; σ^2^_AD_ = direct genetic variance; σ^2^_AS_ = social genetic variance; σ_ADS_ = direct-social covariance; σ^2^_C_ = variance due to common rearing environment; σ^2^_TANK_ = variance of experimental tank; σ^2^_TVB_ = variance of the total breeding value; σ^2^_P_ = phenotypic variance; r_AS,AD_,T^2^ = σ^2^_TVB_/σ^2^_P_; ^2^bounded to zero.

For length of the four fins, the likelihood ratio tests for including SGE were highly significant at recordings 2 and 3. As expected, SGE for length were non-significant at recording 1 for the first and second dorsal fins and for the caudal fin. Contrary to expectations, SGE were significant for length of the third dorsal fin at recording 1 (Table 6). Especially at recording 3, the estimated ratio of total heritable variance over phenotypic variance (T^2^) was high, sometimes significantly greater than 1, although the estimates were inaccurate. The variance due to common rearing environment prior to the recording period was zero or non-significant for all four fins and all three recordings.

**Table 6:** Estimates and standard errors of parameters from a model with social genetic effects for fin length at three recording times

		**Fin**			
**Recording**	**Parameter**^**1**^	**First dorsal**	**Second dorsal**^**3**^	**Third dorsal**	**Caudal**
**1**	LogL	2.12 (0.12)		1.23 (0.29)	0.04 (0.96)
	σ^2^_AD_	7.19 ± 2.49		2.21 ± 0.07	3.30 ± 0.65
	σ^2^_AS_	0.007 ± 0.04		2.3 × 10^-4^ ± 8.3 × 10^-6^	0^2^
	σ^2^_ADS_	0^2^		0.023 ± 0.0008	0.002 ± 0.028
	σ^2^_C_	0.48 ± .73		0^2^	0^2^
	σ^2^_TANK_	0.21 ± 0.11		0.05 ± 0.05	0.21 ± 0.10
	σ^2^_TBV_	6.93 ± 2.75		3.24 ± 0.11	15.59 ± 2.56
	σ^2^_P_	10.22 ± 0.80		4.67 ± 0.15	3.38 ± 1.07
	r_AS,AD_	−0.41 ± 2.21		0.98 ± 0.00	8.61 ± .41
	T^2^	0.68 ± 0.24		0.52 ± 0.07	0.55 ± 0.07
**2**	LogL	7.07 (< 0.001)	6.99 (< 0.001)	10.0 (< 0.001)	4.30 (< 0.001)
	σ^2^_AD_	9.45 ± 1.62	1.31 ± 0.29	1.40 ± 0.52	3.36 ± 0.68
	σ^2^_AS_	0.014 ± 0.005	0.003 ± .001	0.004 ± 0.002	0.001 ± 0.001
	σ^2^_ADS_	0.03 ± 0.06	0.03 ± 0.01	0.03 ± 0.02	0.004 ± 0.06
	σ^2^_C_	0^2^	0^2^	0.09 ± 0.17	0^2^
	σ^2^_TANK_	0.014 ± 0.24	0^2^	0^2^	0^2^
	σ^2^_TBV_	16.36 ± 3.43	3.46 ± 0.69	4.62 ± 1.04	5.69 ± 1.09
	σ^2^_P_	12.12 ± 0.85	4.58 ± 0.19	4.89 ± 0.21	7.84 ± 0.38
	r_AS,AD_	0.10 ± 0.18	0.43 ± 0.26	0.46 ± 0.25	0.79 ± 0.65
	T^2^	1.35 ± 0.27	0.76 ± 0.14	0.94 ± 0.20	0.73 ± 0.12
**3**	LogL	23.91 (< 0.001)	15.06 (< 0.001)	36.29 (< 0.001)	6.96 (< 0.001)
	σ^2^_AD_	6.13 ± 2.06	1.13 ± 0.57	1.76 ± 0.42	4.29 ± 0.88
	σ^2^_AS_	0.03 ± 0.01	0.05 ± 0.04	0.03 ± 0.01	0.013 ± 0.005
	σ^2^_ADS_	0.23 ± .08	0.03 ± 0.01	0.05 ± 0.04	0.05 ± 0.05
	σ^2^_C_	0.51 ± 0.64	0.09 ± 0.20	0^2^	0^2^
	σ^2^_TANK_	0.15 ± 0.40	0.31 ± 0.29	0^2^	0.15 ± 0.21
	σ^2^_TBV_	28.43 ± 6.60	13.84 ± 3.46	15.59 ± 2.56	7.69 ± 2.19
	σ^2^_P_	11.41 ± 0.77	6.24 ± 0.31	6.73 ± 0.28	10.95 ± 0.53
	r_AS,AD_	0.53 ± 0.17	0.26 ± 0.22	0.22 ± 0.18	−0.21 ± 0.18
		T^2^	2.49 ± 0.54	2.22 ± 0.57	2.31 ± 0.35	0.70 ± 0.20

^1^LogL = change in Log likelihood relative to the traditional animal model; P-values are given in parentheses;

σ^2^_AD_ = direct genetic variance; σ^2^_AS_ = social genetic variance; σ_ADS_ = direct-social covariance; σ^2^_C_ = variance due to common rearing environment; σ^2^_TANK_ = variance of experimental tank; σ^2^_TVB_ = variance of the total breeding value; σ^2^_P_ = phenotypic variance; r_AS,AD_,T^2^ = σ^2^_TVB_/σ^2^_P_; ^2^bounded to zero; ^3^the analysis did not converge.

For fin erosions, the likelihood ratio tests for existence of SGE were significant for the second and the third dorsal fins (Table 7) but not for the first dorsal fin and the caudal fin. The variance due to common rearing environment was small and non-significant for all fins, except for the caudal fin, whereas the experimental tank effect was significant for all fins and explained from 9.8 (first dorsal fin) to 24.1% (second dorsal fin) of the total variation in fin erosions. The ratios of total heritable variance over phenotypic variance were high for the second (1.37) and the third (0.48) dorsal fins and much higher than the heritability estimated using the traditional animal model (Table 4).

**Table 7:** Estimates and standard errors of parameters from a model with social genetic effects for fin erosion traits

	**Fin**			
**Parameter**^**1**^	**First dorsal**	**Second dorsal**	**Third dorsal**	**Caudal**
LogL	1.87 (0.15)	10.53 (< 0.001)	44.22 (< 0.001)	0.56 (0.57)
σ^2^_AD_	176.95 ± 40.77	16.44 ± 3.82	0.09 ± 1.08	3.54 ± 4.54
σ^2^_AS_	0.07 ± 0.09	0.19 ± 0.10	0.03 ± 0.03	0.05 ± 0.05
σ_ADS_	1.79 ± 1.19	0.54 ± 0.44	0.04 ± 0.09	0.09 ± 0.23
σ^2^_C_	0.88 ± 9.64	0^2^	0.71 ± 0.55	4.71 ± 2.19
σ^2^_TANK_	20.90 ± 7.06	20.26 ± 6.53	6.05 ± 1.81	8.10 ± 3.24
σ^2^_TBV_	276.0 ± 76.73	114.9 ± 47.57	13.60 ± 10.72	28.02 ± 22.28
σ^2^_P_	213.7 ± 16.80	84.14 ± 5.84	28.36 ± 1.65	64.3 ± 3.26
r_AS,AD_	0.51 ± 0.47	0.30 ± 0.25	0.78 ± 0.44	0.21 ± 0.56
T^2^	1.29 ± 0.33	1.37 ± 0.60	0.48 ± 0.39	0.43 ± 0.36

^1^LogL = change in Log likelihood relative to the traditional animal model; P-values are given in parentheses; σ^2^_AD_ = direct genetic variance; σ^2^_AS_ = social genetic variance; σ_ADS_ = direct-social covariance; σ^2^_C_ = variance due to common rearing environment; σ^2^_TANK_ = variance of experimental tank; σ^2^_TVB_ = variance of the total breeding value; σ^2^_P_ = phenotypic variance; r_AS,AD_,T^2^ = σ^2^_TVB_/σ^2^_P_; ^2^bounded to zero.

### Correlations between direct and social breeding values

Estimates of correlations between direct breeding value for weight at recording 3 and the social breeding values for fin erosions and fin length were negative for all evaluated traits, except for erosion of the second dorsal fin (Table 8). However, all estimates had large standard errors and were not significantly different from zero. The direct genetic effects of weight and of fin length and fin erosion at recording 3 were moderately to highly correlated (0.41-0.85), with the exception of erosion of the first dorsal fin, which was not significantly different from zero.

**Table 8:** Estimates and standard errors of correlations between direct and social breeding values for weight at recording 3 and fin damage traits

		**Social**				
	**Direct**	***Erosion***	***Fin length recording 2***		***Fin length recording 3***	
**Direct**	**Weight**	**Second dorsal**		**Second dorsal**	**Third dorsal**	**First dorsal**	**Caudal**
Weight		0.28 ± 0.23		−0.05 ± 0.24	−0.06 ± 0.24	−0.35 ± 0.20	−0.34 ± 0.20
Erosion second dorsal	0.59 ± 0.22	0.33 ± 0.26					
*Fin length recording 2*							
Second dorsal	0.41 ± 0.13			0.40 ± 0.29			
Third dorsal	0.77 ± 0.12			0.42 ± 0.25			
*Fin length recording 3*							
First dorsal	−0.23 ± 0.26				0.41 ± 0.17		
Caudal	0.85 ± 0.09					−0.38 ± 0.17	

## Discussion

### Estimated genetic parameters for direct and social genetic effects

In this study, we estimated genetic parameters for direct and social genetic effects for growth traits and fin damage traits in Atlantic cod. To our knowledge, this is the first study that investigates the SGE of traits directly related to welfare in aquaculture species. The estimated ratio of total heritable variance over phenotypic variance (T^2^) was significantly greater than 1 for some of the fin damage traits, although the estimates had large standard errors. This means that we documented strongly significant SGE for fin damage traits but that the experiment was not large enough to accurately quantify the total heritable variance, although the experimental design was based on the simulation study by Ødegård and Olesen [18]. They showed that, over an average of 50 replicates, this design would provide accurate breeding values and estimates of direct and social genetic variance components. In addition, Ødegård and Olesen [18] did not fit the family effect (the effect due to common rearing of the families), which we did in the current study and this added an additional parameter to estimate.

The likelihood ratio tests for including the SGE for fin length were highly significant at recordings 2 and 3 for all four fins but estimates of the social additive genetic variances (Var(A_s_)) were small. At first glance, the fact that small estimates for Var(A_s_) are highly significant suggests a contradiction. However, the full social effect caused by an individual equals (n-1)A_s_ and its variance equals (n-1)^2 Var(A_s_), which was large for the cases in which the SGE was significant. Thus, for the significant results, the variance of total SGE is large, and there is no contradiction.

The traditional animal model without SGE was the best model for fin length at initial stocking of the fish (recording 1), whereas the model with SGE was the best model at recordings 2 and 3 (Table 6). This is as expected since the social hierarchy is established in the period after stocking the fish in the tanks. Thus, at recording 1 we expect neither tank effects, nor SGE. Tank effects and SGE were included in the model at recording 1 as a check of our methods. Evidence of large SGE or tank effects at recording 1, would have indicated a problem with our model or experiment. This check of presence of social and tank effects at recording 1 increases the confidence that the observed significant effects at recordings 2 and 3 are real. This check, however, gave one unexpected result, which was the significance of SGE for length of the third dorsal fin at recording 1 (Table 6). This may be the result of the common environment effect mistakenly being detected as a SGE. Our design was not well suited for the detection of the common environment rearing effects since we only had a limited number of half-sib groups. In addition, the estimate of the experimental tank effect converged to zero, indicating that standard errors of the estimates may be underestimated. Nevertheless, the estimates of the variance of SGE at recording 1 were very small and, as mentioned earlier, the likelihood ratio test showed that the conventional animal model was the best model for all four fins at recording 1.

None of the estimates of the correlations between direct breeding values for growth at recording 3 and social breeding values for fin erosions and fin length (Table 8) were significant. Hence, although most estimates were negative, we cannot make any conclusion on whether selection on direct breeding values for growth will result in a reduction in welfare-related traits.

In contrast to fin damage traits and despite the significantly restricted feeding used in the study, no significant SGE were found for any of the studied growth traits. Rutten et al. [16] ran a six-week experiment with 450 fish from eight full-sib families randomly distributed into 45 tanks. Body weight of the fish was recorded at the start of the experiment and at the end of the experiment. No SGE for body weight was found. However, a design with only eight families, eight tanks and a random distribution of fish within each family, as that used in their study, is not optimal to detect SGE [18,23].

Estimates of heritability obtained for growth using the traditional animal model are in line with earlier estimates for Atlantic cod juveniles at tagging age [24,25]. One unexpected result is the differences in heritabilities between the four different fins that were observed for both fin length and fin erosion; for the first dorsal fin, estimates of heritability were extremely high for both length (0.60 to 0.80) and erosion (0.83), which may indicate the existence of a genetic deformity rather than fin damage on the first dorsal fin. To our knowledge, there are no previous estimates of heritabilities of fin length and fin erosions in aquaculture.

In the study by Hatlen et al. [26] on 55 g cod juveniles, the fin damage decreased from the first fin to the second fin, to the third dorsal fin, and was lowest for the caudal fin. This agrees with our study, except that we observed more fin erosions on the caudal fin compared to the third dorsal fin. Hatlen et al. [26] studied growth and fin damage of both dorsal and pectoral fins in three groups of Atlantic cod (55, 250, and 450 g) under feed deprivation. Incidence of fin damage differed between groups; in the 55 g group, which is closest in size to the fish of our experiment, incidences of damage on the dorsal fins were higher than for the other two groups. Pectoral fins, in contrast, were more often damaged in the 250 g fish. In our study, we only considered fin damage of the dorsal and the caudal fins but studies on bigger fish should also consider the pectoral fins, since incidence of fin damage differs between size groups of fish [26]. Hatlen et al. [26] found that fast-growing fish also had a lower incidence of fin damage than slow-growing fish, which suggests that the fish that received most aggression may have been prevented from feeding.

### Methods for assessment of fin erosion

In this study, we used subjective scoring of fin erosions and fin length using digital image analysis to quantify fin damage. Subjective scoring of fin erosion has been done in previous studies for other species, e.g. rainbow trout (*Oncorhynchus mykiss*) [27]. Fin damage can affect growth, survival and welfare of the fish [3,4]. The likelihood ratio tests for including SGE for fin length were highly significant at recordings 2 and 3 for all four fins. In contrast, SGE for fin erosion were only significant for the second and third dorsal fins. Measuring fin length and scoring fin erosion are two ways of quantifying fin damage. For fin length, the maximum length of each fin is estimated, which is phenotypically positively related to body size for fish with undamaged fins [28]. However, fin damage may also occur at the upper part of the fins, which would not affect maximum length of the fin but would be included when quantifying fin erosion. The differences in scoring fin damage based on fin length versus fin erosions are also reflected in the phenotypic correlations between the two traits at recording 3, which were −0.83, -0.89, -0.65, -0.83 for the first, second, and third dorsal fins and for the caudal fin, respectively (He, Nielsen and Olesen, unpublished data).

One advantage of image analysis to measure fin length is that the photos can be analysed after completion of the experiment, which minimizes the handling time of the fish. In addition, for breeding experiments with many fish and families, it is important to have a fast and efficient method to quantify fin damage. However, measuring fin length precisely on large numbers of fish can be challenging. Results from a study of a sub-set of the data used here [29] showed that repeatability estimates for fin length scored by three different persons ranged from 0.46 for the first dorsal fin to 0.61 for the caudal fin. In spite of these relatively low to moderate repeatabilities, significant estimates of heritabilities and social genetic variance were found for fin length in the current study, which indicates that fin length can be used to quantify fin damage in Atlantic cod. However, the method used to measure fin length could be improved by having all digital analyses done by the same person or by using chromatic pictures [29].

### Practical implementation

In this study, we used cod juveniles of about eight months of age (35 g). Usually, farmed Atlantic cod are transferred to sea cages at approximately one year of age, where they are then reared until harvest. The breeding goal in Atlantic cod is weight at harvesting. Thus, one may argue that social interactions should also be studied at a later period. However, detecting SGE during the grow-out period would require recording weight and fin damage traits on a large scale in a design with many small cages in the sea [18,30], which is very demanding in practice. In addition, earlier studies suggested that fin damage [26] and cannibalism are more pronounced in smaller fish [2]. At present, we do not know if our results are also valid for cod at later stages or under less restricted feeding, i.e., whether the genetic correlations between social effects at different ages and with *ad libitum* feeding are high. The current data structure included relatively small groups of fish (21 per tank), which are far from real commercial conditions. When fish are reared in sea cages, group sizes increase sustantially and it is likely that the social hierarchy is less stable in larger groups of fish. In addition, the social effect of a single individual on other individuals may depend on group size, since the social effects are distributed over more animals in larger groups, which is called dilution [30], and because larger groups are typically spread over a larger water volume.

## Conclusions

Based on the results from this study, we conclude that social effects on fin length and fin erosion contribute to heritable variation for those traits under restricted feeding, which is hidden in a classical animal model. Considering estimates of social breeding values for fin length or fin erosion when selecting fish will enable us to improve response to selection in traits related to welfare such as fin length and fin erosion in Atlantic cod juveniles. However, testing for SGE on a large scale would represent a challenge in practice. Further studies are needed to quantify the SGE more precisely and to test whether our results are also valid for the grow-out period.

## Competing interests

The authors declare that they have no competing interests.

## Authors’ contributions

HMN participated in planning the experiment, carried out the statistical analyses, and drafted the manuscript. BBM participated in planning and carrying out the experiment, did part of the digital image analysis and edited the data. PB and JØ participated in planning the experiment and helped with the statistical analysis. HT coordinated and planned the experiment. BD participated in the planning and the analysis of the fin data. IO planned and coordinated the study. All authors were involved in the discussion of results and the content of the manuscript. All authors have read and approved the final manuscript.
